# Understanding Variations in the Tracking and Erosion Performance of HTV-SR-Based Composites due to AC-Stressed Aging

**DOI:** 10.3390/polym13213634

**Published:** 2021-10-21

**Authors:** Rahmat Ullah, Muhammad Akbar, Nasim Ullah, Sattam Al Otaibi, Ahmed Althobaiti

**Affiliations:** 1 Faculty of Electrical Engineering, Ghulam Ishaq Khan Institute of Engineering Science and Technology, Topi 23640, Pakistan; akbar@giki.edu.pk; 2 Department of Electrical Engineering, Taif University, Taif 26571, Saudi Arabia; nasimullah@tu.edu.sa (N.U.); srotaibi@tu.edu.sa (S.A.O.); ahmed.althobaiti@tu.edu.sa (A.A.)

**Keywords:** HTV-SR, silica, ATH, tracking, erosion, multi-stress

## Abstract

Among the polymeric family, high-temperature-vulcanized silicone rubber (HTV-SR) is the most deployed material for high voltage insulation applications. However, in an outdoor environment, due to contamination and wetting-induced dry band arcing, consequently SR experiences surface tracking and erosion. From a practical standpoint, the tracking and erosion performance under multi-stress aging is required to be known. It is in that context that the present study was undertaken to measure and analyze the effect of multi-stress aging on tracking and erosion performance. Composite samples of SR having different filler concentrations of silica and alumina trihydroxide (ATH) were aged in a multi-stress chamber for a period of 5000 h, and after that their electrical tracking performance was studied. Simultaneously, unaged samples were also exposed to tracking test for comparison. To conduct this test, the inclined plane testing technique was used in accordance with IEC-60587. All samples exposed to tracking test were analyzed using different diagnostic and measuring techniques involving surface leakage current measurement, Fourier transform infrared spectroscopy (FTIR), thermal stability and hydrophobicity classification. Experimental results shown that the tracking lifetime increased through incorporation of silica and ATH fillers in the SR. Amongst all test samples, two samples designated as filled with 2% nano silica and 20% micro silica/ATH exhibited greater resistance to tracking. This was attributed to the optimum loading as well as better dispersion of the fillers in the polymer matrix. The presence of nano-silica enhanced time-to-tracking failure, owing to both improved thermal stability and enhanced shielding effect on the surface of nanocomposite insulators.

## 1. Introduction

Insulators made of silicone rubber (SR) insulators are considered an attractive alternative to conventional ceramics insulators due to their superior hydrophobic nature, excellent contamination flashover performances and lightweight etc. [1,2,3]. The SR matrix consists of a repeating polydimethylsiloxane unit, which comprises backbone inorganic siloxane. Each siloxane is further connected with two methyl groups. The existence of inorganic siloxane in SR provides thermal stability, as compared to other polymer matrices, and thus makes it a perfect material to resist different environmental stresses. The methyl group in the structure of SR, being organic and insulating, renders property of hydrophobicity. Owing to its chemical structure, silicone rubber exhibits low surface energy which is an important feature to retain good hydrophobicity. The existence of carbon in the methyl group, however, is more vulnerable to alienation due to the synergistic effect of different stresses when it is deployed in the outdoor environment [4]. The breakdown of methyl groups releases hydrogen and carbon atoms on the surface of SR. When it is exposed to an electrical stress, these carbon atoms form a track, and the phenomena is called tracking. Moreover, in a contaminated environment, wetting induced dry band arcing (DBA), which deteriorates the SR materials and ultimately leads to surface tracking and erosion. It is evident from different studies, that a contamination layer, on becoming wet, can easily form a conductive layer to let the current flow on its surface and eventually induces DBA. Persistent DBA activities cause ohmic heating thus producing abrupt temperature rise and as a result surface of the material gets eroded [5]. Such like effects are of great concern to power utility companies in the context of the long-term life of polymeric insulators. Different standardized test methods have been adopted to know their ability to resist environmental stresses and to measure the degree of aging. One of those is the inclined plane test (IPT) which is standardized through IEC 60587. This test helps to identify tracking and erosion resistance of the polymeric material against surface discharges [6]. In this test, liquid contaminant is let to flow on the surface of electrically-stressed test sample and discharges take place.

Pristine SR has low thermal conductivity, having less heat dissipation capacity, which may cause tracking and erosion and ultimately leads to the failure of insulating material. Studies are, therefore, needed to improve tracking and erosion resistance of polymeric material. Some studies have been conducted over the last three decades in an attempt to suppress the occurrence of tracking and erosion by incorporating micro and nano-fillers of different concentrations in SR.

Hydroxide fillers, such as ATH, enhance resistance to tracking and erosion significantly. In the event of DBA, the temperature of insulator surface may rise to very high values [7,8]. Such rise of temperature erodes the surface and leads to track formation. These spots on insulator constitute high-stress regions with decreased hydrophobicity. The ATH contains water of hydration which, upon release, cools the surface which is at an elevated temperature. Similarly, alumina contained in the ATH is a highly resistive which improves performance. However, beyond an optimum quantity of ATH loading, more hydrates are liberated, which results in lowering tensile strength and also leads to enhanced surface roughness [9].

The effect of different sizes of ATH particles on the performance of RTV-SR-coated insulators was studied by Deng et al. [10], and reported that micro composites with filler sizes of 4.5 and 13 μm exhibited lower leakage current which caused minimum effect on surface roughness. Kemaloglu and co-workers [11] reported improvement in different properties of SR insulators filled with nano/micro boron nitride. The dielectric strength of the SR-filled samples was found higher in comparison with unfilled samples. The SR nano-composites exhibited superior performance than micro-composites at various investigated levels of doping. Thermal conductivity of filled SR increased by about 10 times to that of unfilled neat SR. Du et al. [12] investigated the influence of loading various nano particles of boron nitride on the tracking and erosion resistance of the RTV-SR, and a relationship was proposed between thermal conductivity and the tracking resistance. The findings also showed a significant improvement in thermal dissipation and conductivity with loading of fillers. Erode mass and DBA decreased thus enhancing the tracking of SR-boron nitride composites. Polymer hybrids are also used as electrical insulation. These composites can be prepared by dispersing both nano/micro fillers in the same polymer matrix. The authors in [13] evaluated the effect of inorganic fillers on tracking and erosion resistance of nano-composites. Micro/nano fillers of both ATH and SiO_2_ were used and the ATH-filled composites showed more resistance to erosion and tracking. Fabiani et al. [14] studied the silica-filled micro-composites, nano-composites and hybrid micro/nano epoxy composites under AC and DC stresses. Tariq et al. [15,16,17,18] have recently studied the effect of BN, silica and aluminum nitride on the tracking and erosion performance of SR/EPDM, and noticed that addition of BN enhanced tracking and erosion resistance as compared to the other fillers.

From the literature review summarized in this section, it has become known that fillers play an important role in the tracking and erosion properties of polymeric insulators. However, up till now, most of the literature discuss variations in the tracking and erosion performance of the SR due to filler loading, and knowledge related to the synergistic effect of multiple stresses (UV, heat, acidic rain, moisture and electric stress) on the tracking/erosion resistance of SR composites is very sparse. In addressing that issue, the present research has been devoted to see the synergistic effect of all important environmental parameters representing a truly harsh environment of Karachi, Pakistan, through 5000 h of aging in a weathering chamber under AC stress. The tracking and erosion resistance of the aged and the unaged samples were measured based on the IEC 60587 [6]. Different diagnostic techniques, such as thermal imaging, Fourier transform infrared spectroscopy (FTIR) and leakage current, were employed in this study.

## 2. Materials and Methods

### 2.1. Fabrication of Test Samples

SR was obtained from Lanxess chemicals. Fillers such as micro/nano-silica (5 µm/12 nm) and ATH were procured from Sigma Aldrich, Darmstadt, Germany, and Degussa chemicals, respectively. The SR gums were mixed initially by using a two-roll-mill. Then, fillers, before loading, were kept in an oven for 12 h to be fully dehydrated. After that, SR gums were mixed with fillers with the help of the two-roll-mill. Varox-organic-peroxide, a curing agent, was applied to the mixture and allowed to blend for another 45 min. Curing of the test samples was done in two stages. Initially, the mixture was pressed at 10 MPa and kept at a temperature of 170 °C for 10 min. Therefore, the samples were cured for 10 min at 200 °C. Samples with six different concentrations were fabricated as shown in Table 1.

### 2.2. Experimental Arrangement for Aging

Design of multi-stress aging parameters was carried out in line with a method proposed by Electric Power Research Institute (EPRI), USA for aging. Harsh environmental conditions of Karachi, Pakistan, were incorporated to design the weather cycle to apply for the present aging study. The samples were energized at 4 kV AC representing a creepage distance of 25 mm/kV. Detailed information about the chamber and the design of the weather cycles were given in our publications [19,20]. 

### 2.3. Inclined Plane Testing (IPT)

**Figure 1:**
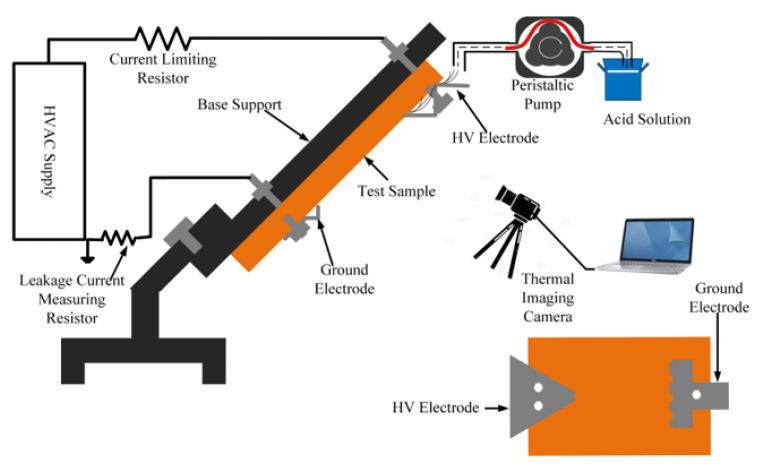
Arrangement for incline plane testing.

**Figure 2:**
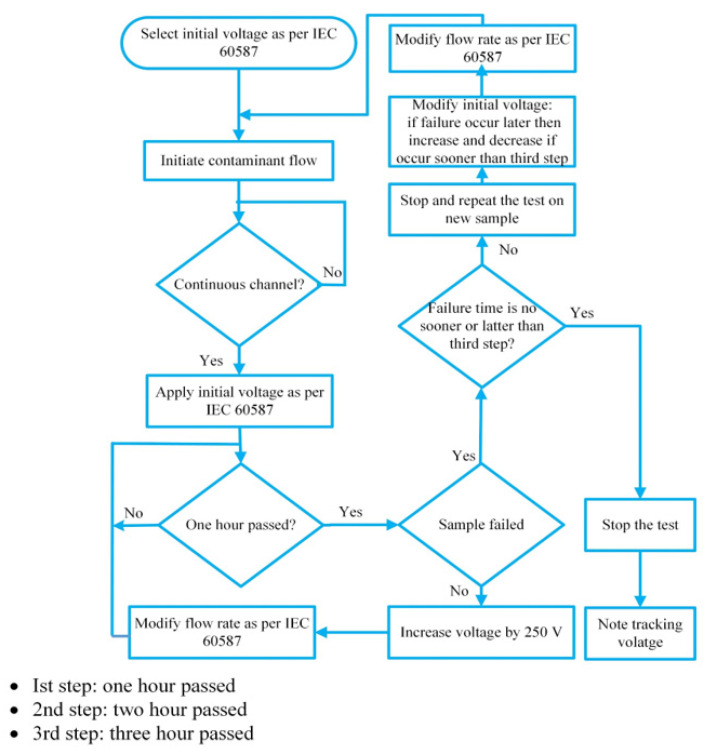
Flow chart of inclined plane testing (IPT).

For the IPT, a protocol specified in IEC-60587 was used [6]. Figure 1 gives a schematic diagram of the experimental arrangement. A tank type AC test transformer system procured from Himalayal China. Moreover, the system also consists of regulating transformer, AC test transformer, power line filter, compensating reactor, coupling capacitor/HV-divider, switch gear cabinet, measuring system and control unit. The output voltage varied between 0–30 kV and the current rating is one ampere. The test sample was positioned at an angle of 45° on an inclined plane. The two electrodes were installed out at each side of the test sample. To ensure smooth flow of liquid contamination on the sample, five layers of filter paper were also inserted into the upper high voltage electrode. The contaminant solution’s conductivity was maintained at 2.5 mS/cm The desired conductivity was achieved by using HANNA HI2211 PH/ORP meter. In compliance with the IEC standards, the flow rate was maintained at 0.15, 0.225 and 0.30 mL/min at voltage level of 2, 2.75 and 3 kV, respectively. The desired flow rate was maintained by shenchen precision pump, with a flow rate ranging from 0.0024–190 mL/min. As per standard, voltage was applied in an hourly step-increase of 250 V. If the test sample failed before the two steps, then the experiments were terminated and repeated with a lower initial voltage. A detailed experimental flow chart is shown in Figure 2. The failure criteria of the sample as per IEC standard specifies that the leakage current exceeds 60 mA for more than 2 s or the tracking length surpasses 25 mm. In this research work, the first criteria was adopted as leakage current detection was much easier and reliable and acceptable internationally.

### 2.4. Diagnostic Techniques

Different measurements were taken on all samples exposed to 5000 h of aging. To determine each type of degradation and its severity. A hydrophobicity test was performed on all the samples using STRI guide. The surface leakage current was measured by knowing the voltage drop using Keithley 182-M sensitive voltmeter across the shunt resistor having value of 20 kΩ on the ground side of the test samples. 

FTIR provides the transmission/absorption spectra of the test samples respecting wave numbers (in cm^−1^) ranging from 500–4000. Each wave number narrates to a specific functional group of the material. The transmission and absorption spectra are basically the reciprocal of each other and so any can be used for the assessment of the test samples. Chemical analysis was performed by Perkin-Elmer spectrum two FTIR spectrometer (PerkinElmer UATR two) before and after the IPT to know the chemical degradation that occurred in each test samples. In this study, samples were directly placed on the crystal (Germanium) of the universal attenuated total reflectance alongside with the 80 unit of pressing force.

Current flow causes a temperature rise due to ohmic heating in every electrical circuit. During IPT, thermal camera FLIR E80 was deployed to take the IR images of the test samples. Camera is capable to measure in the range of FLIR E80 250 °C. The thermal distribution of the samples can be deducted from the IR images of the test samples. Additionally, thermogravimetric analysis of all the unaged, and after the IPT, were performed.

## 3. Results and Discussion

### 3.1. Tracking and Erosion Studies

**Figure 3:**
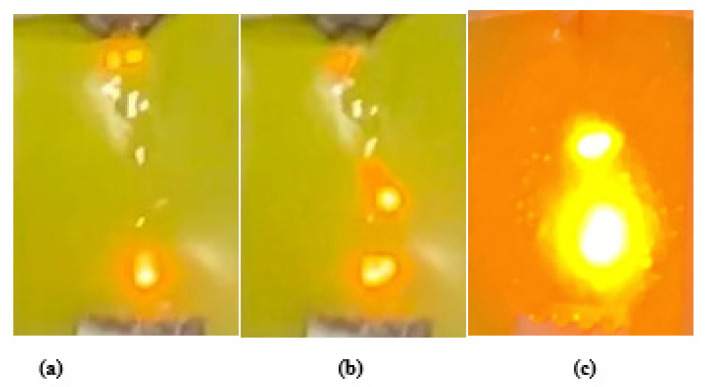
Pictures of the (**a**) initiation of discharges, (**b**) at mid and (**c**) before the end of the experiment.

**Figure 4:**
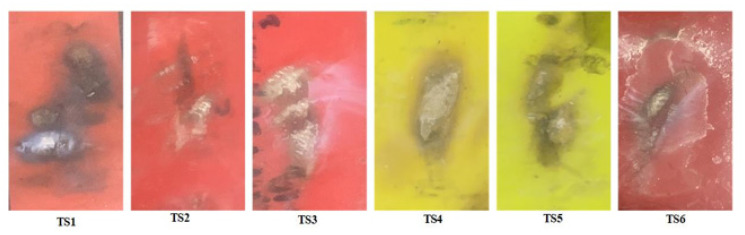
Tracking and erosion pattern of the unaged samples.

**Figure 5:**
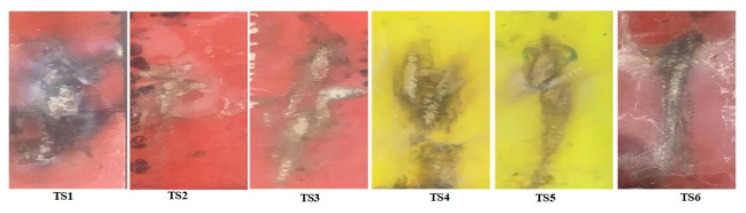
Tracking and erosion pattern of the aged samples.

**Figure 6:**
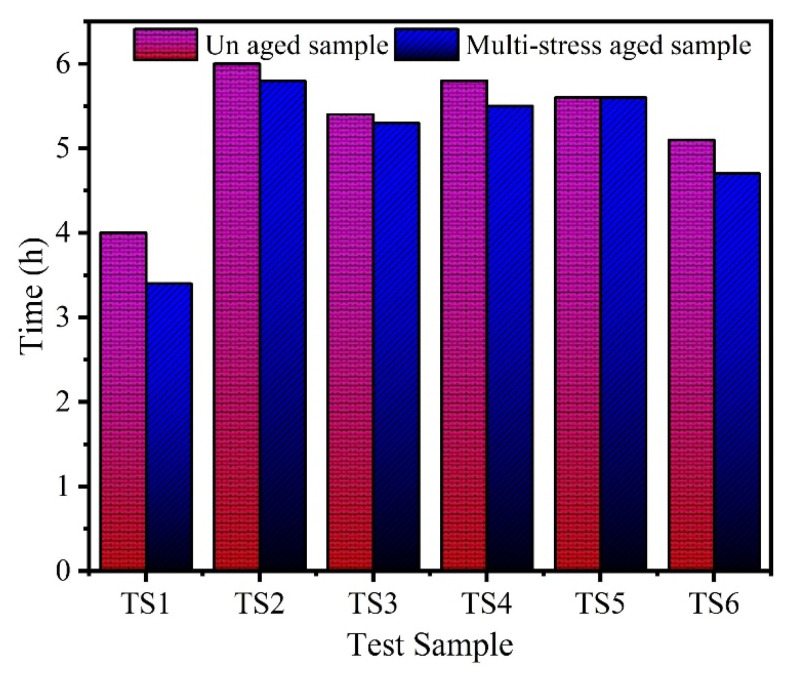
Tracking time of aged and virgin samples.

An effort was made in this study to investigate the effect of multi-stress aging on the tracking and erosion performance of neat HTV-SR and composites loaded with different concentrations of silica and ATH. Firstly, the samples were exposed to multi-stresses representing prevailing environment of Karachi, Pakistan such as heat, UV, humidity, acid rain and electrical stresses for a time span of 5000 h. Due to different stresses, the de-polymerization occurred which reflected some degradation of the test samples. In IPT, leakage current starts flowing due to flow of contaminant on the surface of the test sample which induces ohmic heating. Due to evaporation, dry bands are formed which subsequently leads to DBA. Such DBA are due to a large potential difference. The persistent occurrences of the DBA results in the creation of carbonaceous track and as a consequence, the surface of sample becomes eroded. Figure 3 shows the formation of discharges during the different levels of the IPT. Material erosion, which is accompanied by hot spot formation near the ground electrode, is observed. Heat generated from these hot spots leads to burning and contribute significantly to the enormous erosion of the material. Figure 4 and Figure 5 show the tracking pattern and erosion of the unaged and aged samples after the IPT. From the figures, it is clear that tracking pattern and erosion of the unfilled sample, TS1 is comparative more as compared to the other composite samples. Less tracking and erosion was observed for the test sample, TS2 which is filled with 2% nano-silica. This improvement may be attributed due to loading of the inorganic nano-filler. At higher particle loading of silica however, poor tracking resistance is observed. Due to the synergistic effect of multi-stresses, the samples showed more degradation as compare to the unaged samples. However, the level of degradation in composites samples is lower as compared with the unfilled samples. The nano-fillers appear to form a strong UV shielding layer in the composites that retards the process of degradation. The tracking time of each sample was noted as shown in Figure 6. It can also be observed that the time-to-track was less for the unfilled sample as compared to the other composite samples. 

**Figure 7:**
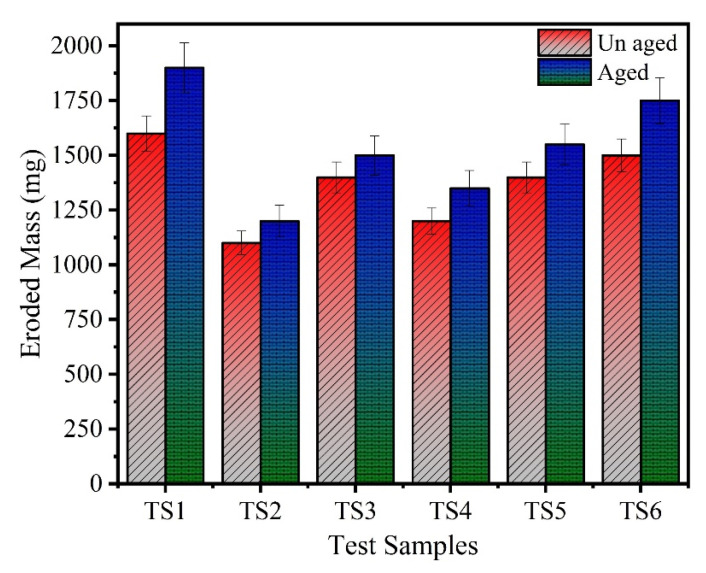
Eroded mass of the unaged and aged samples.

Pictures of the eroded area on both unaged and aged samples after the IPT are earlier shown in Figure 4 and Figure 5. In Figure 7, the relative average change in the mass of the samples is shown with error bars. It can be seen clearly that in the neat (unfilled) sample as well as the 15% nano-silica-filled sample, the eroded mass is substantially greater. Compared to the remaining samples, the samples TS2 and TS4 show some improved resistance to erosion. It may be noticed that this has been attained in hybrid composites by adding only a small quantity of nano-silica. Two percent nano-silica + 20% micro-silica show slightly less erosion among hybrid composites. It is seen from the above results that hybrid composites with only a small concentration of silica are less susceptible to the erosion failure than those filled with nano-silica composites of high concentration.

### 3.2. Surface Leakage Current

**Figure 8:**
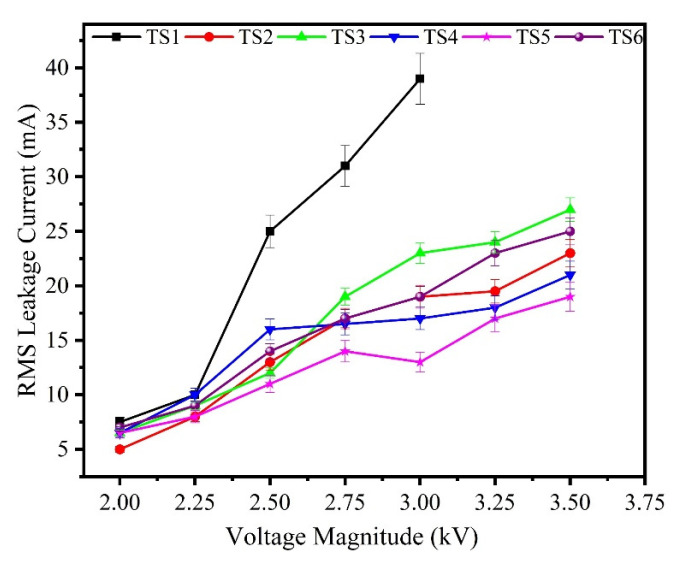
Leakage current of the unaged samples.

**Figure 9:**
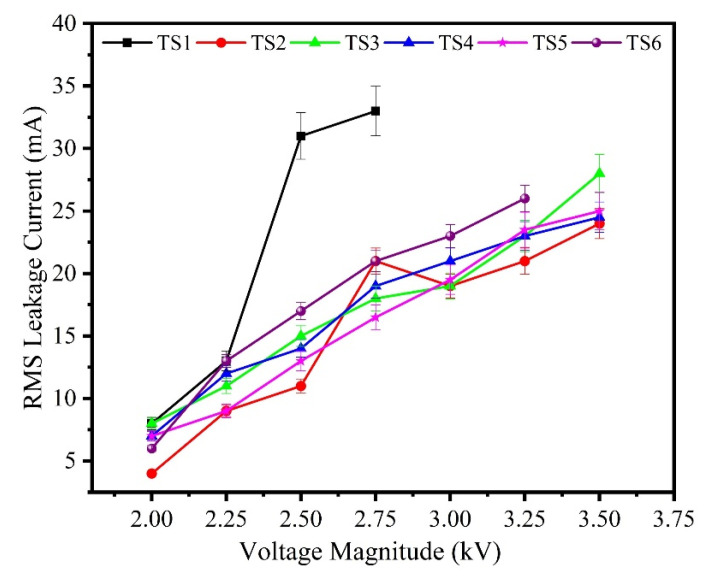
Leakage current of the aged samples.

The results of surface leakage current of the unaged and aged specimens are shown in Figure 8 and Figure 9. It can be observed that leakage current decreases with the incorporation of nano-silica concentration introduced in the base matrix. The shift in charge transport dynamics of polymer composites could explain this phenomenon. The introduction of nano-fillers enhances the concentration of deep traps in polymeric materials, as explained in [21]. The presence of deep traps will impact the dynamics of charge transport, from this perspective, as deep traps lead to a higher capture of mobile charge carriers. As the intensity of nanoparticles introduced increased, the impact of deep traps also increases and then the properties of transport charges would become more apparent. 

Initially, the leakage current in 0 and 30 wt.% of silica is higher to some extent which could be attributed of early hydrophobic loss. With the passage of time, the specimens slowly lost their hydrophobicity and leakage current spikes of higher magnitude were recorded on samples. Compared to sample TS2 and TS4, relatively high spikes of leakage current occurred on samples of 0 and 15 wt.% silica composites. After 3 h, spike amplitudes of leakage current mostly exceeded 20 mA, suggesting extreme DBA activity. Interestingly, the composites of silica/ATH exhibited lower spikes over the specific period of the IPT. These results are consistent with the earlier tracking and erosion results, as explained above.

Hydroxyl and carboxyl groups are known to be formed on the surface of SR during multi-stress aging, which leads to enhanced wettability caused by reduced hydrophobicity. The hydrophilicity of the aged samples is initiated faster than these samples which are not aged. The reduction in hydrophobicity contributes to rapid decomposition of the polymer chain, creating more carbonized path on surface due to IPT. Amongst all samples, the time to failure of the sample designated as TS1 found is much smaller than failure time of other sample as can be seen from Figure 6. Due to non-presence of flame-retardant ATH and silica fillers, the quick failure of sample TS1 is easily understandable.

### 3.3. Hydrophobicity Classifications

**Figure 10:**
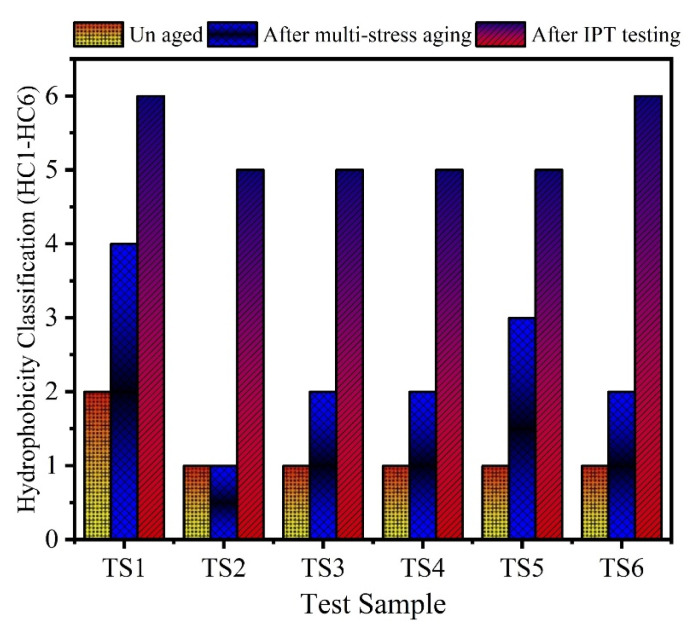
Hydrophobicity classification of the aged and unaged samples.

To assess the loss of hydrophobicity in the materials, experiments were also carried out. Measurements of hydrophobicity were performed by using the STRI guide. According to that guide, hydrophobicity class (HC)-1 is a completely hydrophobic surface, whereas an HC-6 is a completely hydrophilic. The HC of all the unaged and aged samples were performed and results are given in Figure 10, where it can be seen that, when aged samples undergo IPT, all samples experience the highest loss of hydrophobicity. Neat SR (TS1) and TS6 showed the highest permanent hydrophobicity loss followed by other hybrid composites test samples. The decrease in hydrophobicity was caused by quicker polymer chain decomposition that leads toward creation of more surface carbonized paths due to IPT. 

### 3.4. Thermal Analysis

**Figure 11:**
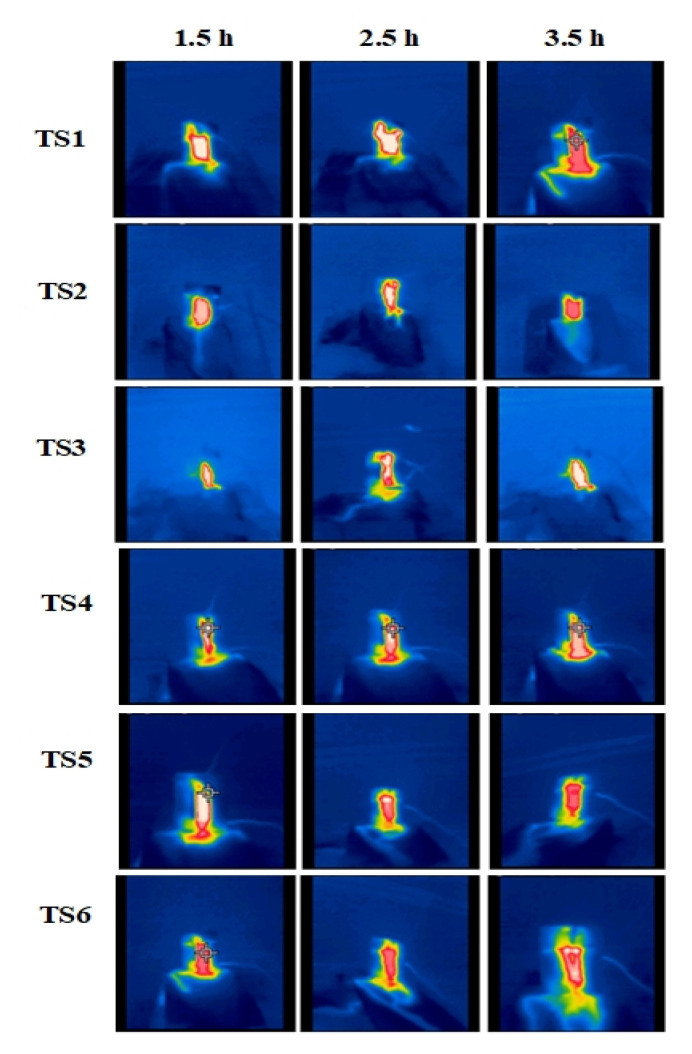
Infrared thermograph of the unaged samples from TS1 to TS6.

**Figure 12:**
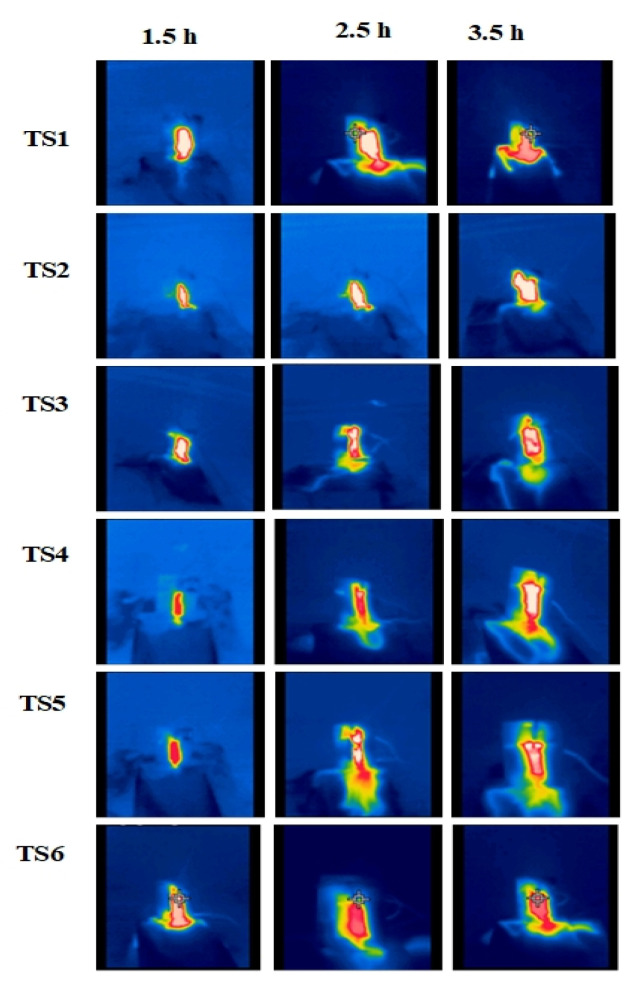
IR images of the multi-stress-aged samples from TS1 to TS6.

Thermal distribution on composites, reported intermittently during the IPT, is shown in Figure 11 and Figure 12 by IR Images. It appears that heat has mostly been amassed in the discharge area due to DBA, which greatly enhances surface temperature. It is evident from the thermographs that the heat is usually extended to the surface of the specimens throughout the IPT. Intense DBA started at 0 and 15 wt.%-silica at 2.5 h during IPT. In a comparative analysis, less heat is amassed in 2 wt.% of the silica and 15 wt.% ATH composites, indicating their improved heat conduction capability in the discharge area. As the IPT proceeds, the arcing region also increased and accumulated most of the area between both electrodes. Comparatively, more discharges occurred in the aged samples as compared with unaged samples. This is attributable to surface degradation which occurred during the 5000 h of multi-stress aging.

**Figure 13:**
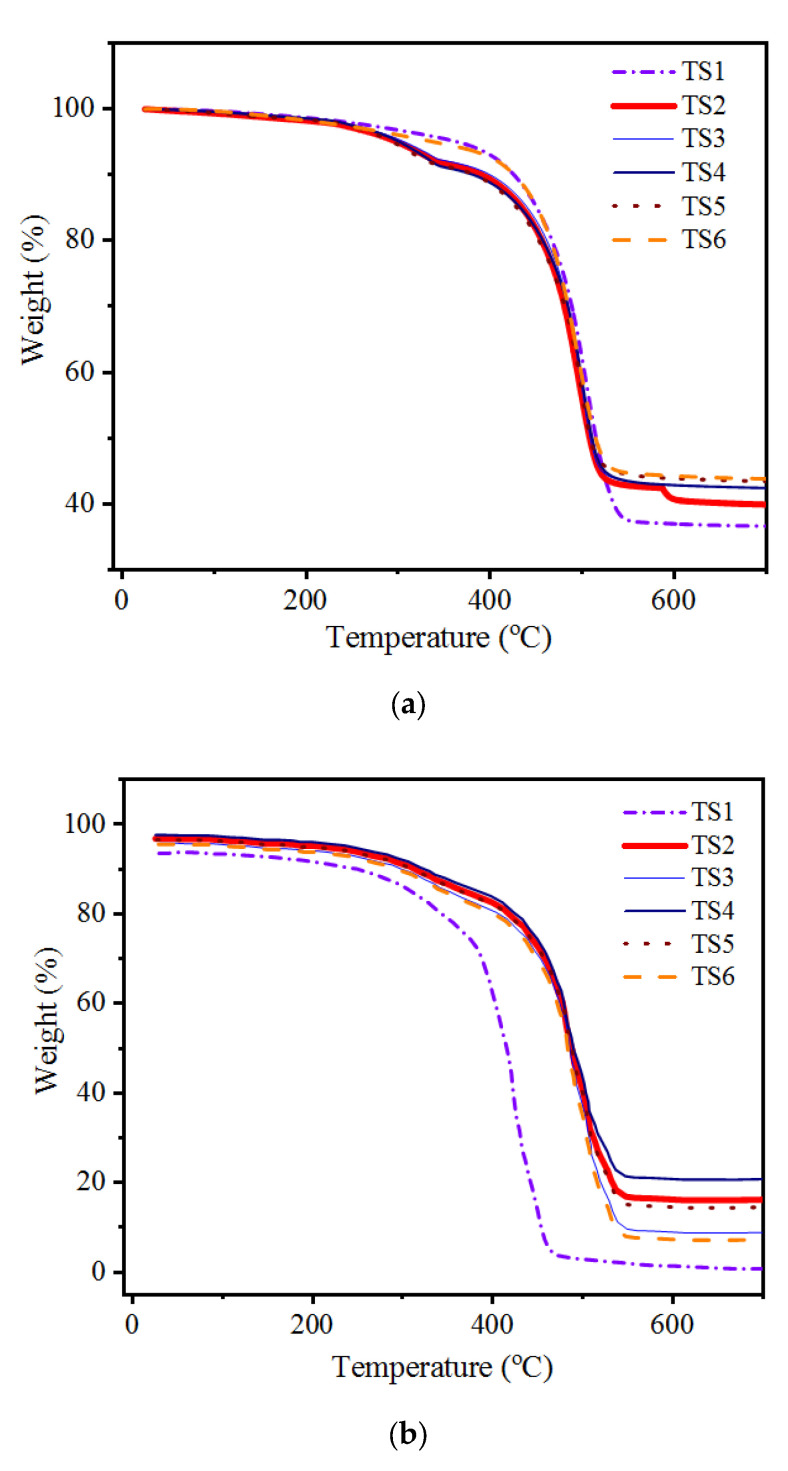
TGA curves of (**a**) unaged and (**b**) aged samples.

**Figure 14:**
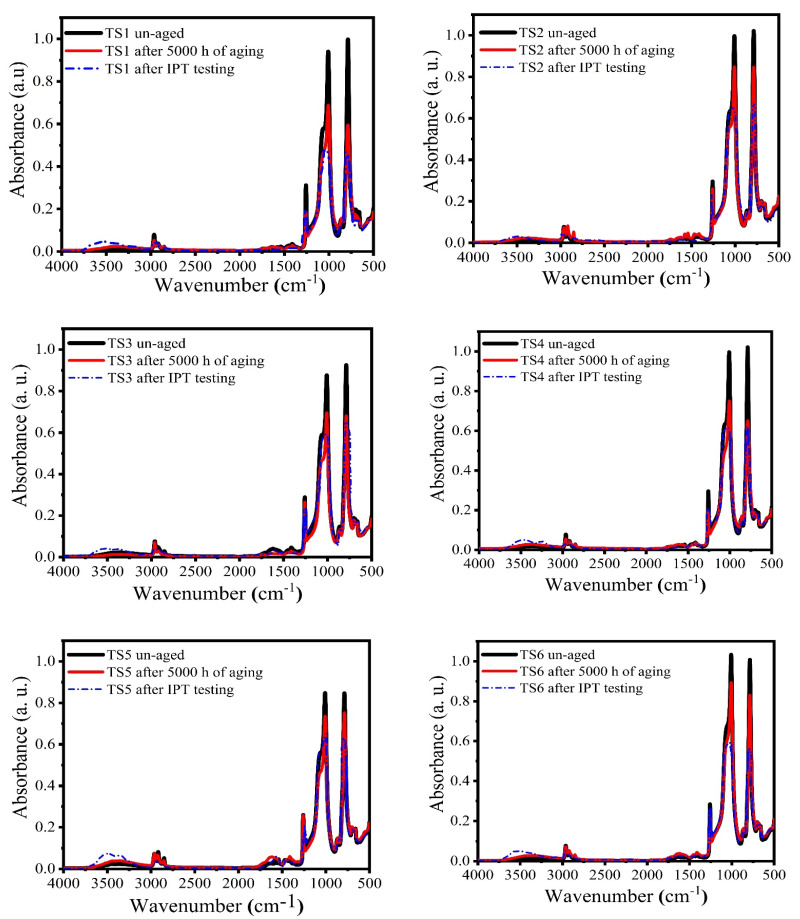
FTIR analysis of the test samples in unaged, aged and aged + IPT conditions.

Due to repeated discharges on the surface, tracking and erosion occurred during IPT mainly caused by thermal accumulation. Therefore, the thermal stability of the composites is expected to have a positive effect on the ability of the specimens to endure DBA-induced tracking and erosion. Figure 13 displays the TGA curves of the unaged and aged samples during IPT. It can be seen that, without any degradation, all the test samples are stable until 220 °C. Initially, with a dip between 220 –350 °C for ATH-filled samples, maybe this is due to the removal of water from ATH particles. Additionally, it should be noted that all significant deterioration began at around 350–400 °C and may be due to the breakage of the hydrophobic methyl (-CH3) side-chain group from the main PDMS chain [22,23,24]. In the case of silica/ATH, all the characteristic temperatures increased relative to other specimens. A very low thermal stability performance is exhibited by the neat SR. The improvement in thermal stability can also be noticed evidently in Figure 14 by linking the curve of the loaded samples and the pure specimens. The better interaction between nano-sized fillers and the SR matrix could explain the increase in thermal stability.

Degradations of the test samples can also be co-related through the TGA curves. The thermal stability of the aged samples was inferior as compared to the unaged sample. In addition, as another indicator for estimating thermal stability, the residual weight at the final 800 °C can be considered. A neat (pure SR) sample had the lowest residual weight among all other samples. The incorporation of ATH and silica results in significantly higher residual weight, particularly for the sample containing of 2% silica by weight. The residual weight of all aged specimens was found to be lower than all unaged specimens. From the findings of thermal stability, it could be inferred that the thermal stability of composites is strongly correlated with resistance to tracking and erosion. Improved thermal stability leads to greater resistance of PDMS against tracking and erosion. Furthermore, the interaction of the particles with the matrix of the PDMS has a strong influence on the thermal stability of the composite.

### 3.5. FTIR Spectroscopy

FTIR measurements were conducted on all aged and unaged samples for comparison and to evaluate the chemical changes on the surface due to both multi-stress aging and IPT. C-Si symmetric stretching at around 788 cm^−1^, asymmetric stretching at 1260 cm^−1^ and Si-O-Si at 1008 cm^−1^ and CH3 at 2960 cm^−1^ with symmetric stretching C-H are the chemical bonds present in the SR of primary interest [25]. As shown in Figure 14, after multi-stress aging and IPT, the FTIR range of the unaged samples are compared with the deteriorated sample. The severity of degradation was determined by evaluating the absorbance peaks of the aged and virgin samples of several chemical groups as shown in Table 2. The decline in absorption peaks due to aging suggests that essential chemical groups in SR and its composites have been degraded. The loss in main chain bonds (Si–O-Si) validates de-polymerization and, as a result, the properties of these materials have deteriorated. 

## 4. Conclusions

Having exposed aged and unaged samples of different formulations to IPT, the main outcomes are summarized here:

1. The 2% level of nano-silica concentration in the HTV-SR performs better as compared to the unfilled and overly loaded composites.
2. Among all test samples, the tracking failure time of the two test samples (TS2 and TS4) were higher as compared to other investigated samples. The tracking resistance was found to decrease due to aging. However, the test samples TS2 and TS4 exhibited higher stability and tracking/erosion resistance among all samples. The correlation also exists with other results, like degradations determined through FTIR spectra hydrophobicity classification and thermal stability analysis. From these results it can be concluded that the sample filled with 2% nano-silica experienced the least degradation among all other samples.
3. The reported results suggest the need of further research on the use of nanoparticles in polymer matrices obtaining more suitable polymeric hybrids, in order to manufacture insulation for electricity transmission distribution systems.

## Figures and Tables

**Table 1:** Fabricated samples with different concentrations.

Test Sample Names	Base Matrix (%)	Nano Silica (%)	Micro Silica (%)	Micro ATH (%)
TS1	100	0	0	0
TS2	78	2	20	0
TS3	72	6	20	0
TS4	78	2	0	20
TS5	72	6	0	20
TS6	85	15	0	0

**Table 2:** Percentage loss of variations in different peaks.

Wavenumber (cm^−1^)	Chemical Groups		TS1	TS2	TS3	TS4	TS5	TS6
788	Si (CH_3_)_2_	Aged	36	9.2	21	11	14.26	16.61
		IPT	54	26	33	35	39	41
1009	Si-O-Si	Aged	26.45	13.71	16.30	13.27	16.26	18.17
		IPT	69	38	44	48	47	51
1255–1270	Si-CH_3_	Aged	33.51	1.78	8.78	1.88	12.96	6.14
		IPT	62	36	41	43	45	48
2960–2963	C-H in CH_3_	Aged	10.13	1.28	1.3	3.51	3.05	3.74
		IPT	39	15	19	21	22	29

## Data Availability

Not applicable.
